# Spillover Effects among Electricity Prices, Traditional Energy Prices and Carbon Market under Climate Risk

**DOI:** 10.3390/ijerph20021116

**Published:** 2023-01-08

**Authors:** Donglan Liu, Xin Liu, Kun Guo, Qiang Ji, Yingxian Chang

**Affiliations:** 1 State Grid Shandong Electric Power Research Institute, Jinan 250003, China; 2 School of Economics and Management, University of Chinese Academy of Sciences, Beijing 101408, China; 3 Institutes of Science and Development, Chinese Academy of Sciences, Beijing 100190, China; 4 School of Public Policy and Management, University of Chinese Academy of Sciences, Beijing 100190, China; 5 State Grid Shandong Electric Power Company, Jinan 250001, China

**Keywords:** electricity price, fossil fuel, carbon emission, climate risk, *HDD*, *CDD*

## Abstract

With the increase in global geopolitical risks and the frequent occurrence of extreme climate in recent years, the electricity prices in Europe have shown large fluctuations. Electricity price has an important impact on the cost of production and living, while electricity demand will also affect other energy markets. A double-layer system based on the spillover effects from a systematic perspective is constructed in this paper to explore the connectedness between different electricity markets and other related energy markets in Europe, considering the impact of climate risks. The results show that there are certain spillover effects among electricity markets in different countries, with a temporary upward trend in the beginning of the Russia–Ukraine conflict, and the electricity markets in the UK and Germany have a more important role in Europe. There are two-way spillover effects between the electricity market and fossil fuel markets, carbon market and carbon emission. Since 2022, the electricity market is affected by gas prices, while it has a certain impact on carbon emissions. The heating degree day (*HDD*) has significant spillover effects on the electricity market and other energy markets, while the spillover effects of the cooling degree day (*CDD*) are relatively small.

## 1. Introduction

Since the Russia–Ukraine conflict, global energy prices have risen rapidly. In response to climate change, European countries have been increasing their share of renewable energy generation to have a clean energy transition, which can further support the aims announced in the Paris Agreement [1]. Although the EU Green Deal calls for climate neutrality by 2050 and emission reductions of 50–55% in 2030 in comparison to 1990, and EU Member States’ Green Deal responses in their final NECPs addressed most of the critical components [2], it will increase the cost of electricity systems [3]. Over 42% of total electricity production was still from fossil fuels by 2021. The overall European wholesale electricity price rose from an average of €35 per megawatt-hour (MWh) in 2020 to above €500 per megawatt-hour (MWh) in 2022 [4]. It has strained the electricity market as most of the traditional energy consumption in Europe relies on imports from Russia. Furthermore, the European wholesale electricity pricing mechanism is the marginal pricing method. The price depends on the final price of electricity according to the cost of production traded in the last unit, which in turn depends on the energy sources used in the electricity generation. The cost of electricity per unit from the renewable energy has fallen rapidly in recent years, but renewable energy generation cannot meet all the electricity demand in Europe. Thus, the rising cost of traditional fossil fuels, especially natural gas generation, has led to a recent surge in electricity prices in Europe [5].

The rapid rise in electricity prices has increased the cost of production as well as the cost of living of households, not only boosting inflation in Europe but also causing a range of social problems. Especially in the season requiring heating, the demand for electricity will rise further. Renewable generation is affected by the current installed capacity and restricted by the climate condition with a high instability. For example, if the wind speed is relatively low, it cannot meet the minimum standard of wind power generation. In addition, the current limited energy storage equipment in Europe also further widens the electricity demand gap. Therefore, the fluctuation of European electricity markets, especially the price of electricity, has now attracted social attention.

The positive impact of fossil fuel prices as well as carbon emission prices on electricity prices has been verified in empirical studies in various countries [6,7,8,9]. Both fossil fuel and carbon emission allowances remain an important part of the cost of power generation worldwide at this stage. In recent years, with countries’ attention to climate change and the development and utilization of renewable energy, the scale and permeability of renewable generation have also played a significant negative impact on the electricity price [10,11,12]. Most of the existing studies analyze and verify the one-way impact mechanism. However, the changes of electricity market and other related markets have a certain spillover effect. With the increase in electricity demand, the demand for traditional fossil fuel and renewable energy will also increase, promoting the rise of energy prices. Similarly, as traditional fossil fuel is still an important part of electricity generation, increased electricity demand will also drive the demand for carbon emissions allowances, thus raising the carbon price. In addition, when electricity prices rise rapidly, the high cost of electricity will also reduce the demand for electricity in the production sectors and households, thus reducing carbon emissions and having a negative impact on carbon prices. Therefore, in the context of violent fluctuations of the energy prices in Europe, it is important to study the dynamic interaction between the electricity markets and other related energy markets to better understand the price transmission mechanism and prevent the energy crisis in Europe.

At the same time, climate risks have an important impact on electricity demand and supply as the impact of extreme climate intensifies in recent years. On the one hand, extreme heat or extreme cold temperature will increase the cooling and heating demand, raising the overall demand for electricity, and it has a positive impact on the electricity price. The Green Deal has increased the power-to-heat in the district heating sector [13]. On the other hand, both wind and solar electricity generation are dependent on climate conditions. Long-term low wind speed or cloudy days will reduce the renewable generation, thus increasing the electricity price. However, current studies of the impact of climate change or climate risks on the electricity market is still limited, and they mostly focus on the impact of cold freezing, ignoring the impact of other climate risks on electricity supply and demand. Therefore, this paper also introduces climate risk to the investigation of the spillover effect between electricity market and other markets, exploring the direct and indirect influence mechanism of different climate factors on electricity price.

The structure of the paper is given as follows. The second section will review the relevant literature and propose the innovation of this paper. The overall methodology and methods as well as the dataset and related indicators will be introduced in the third section. In the fourth section, the dynamic connectedness among electricity prices in the five European countries will be analyzed. Furthermore, the dynamic spillover effects among European electricity prices and fossil fuel prices, carbon prices, carbon emissions, and climate risk factors will be explored and discussed in the fifth section. Conclusions and suggestions will be given in the last section.

## 2. Literature Review

Many studies in the literature focused on the electricity price prediction in the short term, applying a large number of econometric models, machine learning or deep learning models to make a short-term prediction of the electricity price through the fluctuation characteristics of the electricity price itself [14,15,16,17,18,19,20]. However, in these forecasts, less consideration is given to the impact of other energy prices, carbon emissions, and extreme weather on electricity prices and their inherent mechanisms. The electricity price prediction in the long term often takes into account the economic variables, supply and price of various energy sources as well as carbon emission-related policies [12,21,22,23].

With the continuous improvement of the electricity market mechanism, the degree of marketization and openness has been strengthened. The linkage between electricity markets in different regions and other traditional energy as well as renewable energy markets is strengthening, which has also attracted extensive attention from the academic community.

On the transmission relation of electricity prices in different regions, Menezes et al. investigated associations between spot prices from the British, French and Nordpool markets with those in connected electricity markets and fuel input prices and found that British electricity spot prices are associated with fuel prices and not with price developments in connected markets, while the opposite is observed in the French and Nordpool day-ahead markets [24]. Keles et al. examined the interdependencies between the Swiss electricity market and those of neighboring countries and found that the Swiss electricity price correlated strongly with the German electricity price in summer, while it tends to follow the French electricity price in winter [25].

As for the relationship between electricity price and traditional energy prices, a lot of research focuses on the impact of oil price, natural gas price and coal price on electricity price. Emery and Liu analyzed the relationship between electricity futures prices and natural gas futures prices and found that the daily settlement prices of New York Mercantile Exchange’s (NYMEX’s) California–Oregon Border (COB) and Palo Verde (PV) electricity futures contracts are cointegrated with the prices of its natural gas futures contract [6]. Nakajima and Hamori tested the Granger causality-in-mean and in-variance among electricity prices, crude oil prices and yen-to-US-dollar exchange rates in Japan and found that although the exchange rates and oil prices influenced power generation costs, the Granger causality from neither the exchange market nor the oil market to the power market can be found [26]. Gil-Alana et al. conducted a fractional integration and cointegration study, and the results showed that the oil price and interest rate had significant positive effects on the electricity prices in Kenya [7]. Ohler et al. investigated the influence of fuel price volatility on electricity price and found the cola and natural gas costed Granger causality electricity prices for industrial and commercial customers in US states [9]. Kristjanpoller and Minutolo applied a multi-fractal asymmetric detrended cross-correlation analysis to analyze the presence and asymmetry of the cross-correlations between the price of electricity in U.S. with respect to the crude oil and natural gas markets and found that the cross-correlation is higher in the case of oil and electricity pairs than the natural gas pairs [27].

Renewable energy also has a certain impact on electricity price. However, due to the lack of indicators for renewable energy prices, existing studies have mostly discussed the impact on electricity price from the perspective of renewable energy production and penetration. Trujillo-Baute et al. analyzed the degree of influence of RES-E promotion costs on the evolution of electricity price in EU member states, and the results showed that the impact of renewable energy promotion costs on retail electricity prices is positive and statistically significant, although relatively small [10]. Dong et al. studied the impact of penetration of renewable energy on electricity price and found that electricity price was more stable in Sweden as hydropower is a more stable energy source, while in Danshi price areas, the volatility of electricity prices is clearly affected by wind power [28]. Rowińska et al. introduced a four-factor arithmetic model including deterministic seasonality and trend function, which are both short- and long-term stochastic components for electricity baseload spot prices in Germany and Austria, and the empirical results showed that taking into account the impact of the wind energy generation on the prices improves the goodness of fit [11]. Tselika employed the quantiles via moments (MMQR) method to investigate the impact of intermittent renewable generation on the distribution of electricity price and found that the wind generation increased the occurrence of price fluctuations for low demand in both Denmark and German [29]. Schönige and Morawetz studied the influence of renewable production on electricity spot prices in European countries and confirm a U-shaped relationship between the share of renewable electricity production and price variance in seven countries while the minimum price variance for most countries is found to be between 10% and 40% [30].

With the emergence of Emission Trading Systems, carbon price, as an important cost of electricity, also has a certain impact on electricity price. Freitas and Silva evaluated the influence of CO2 opportunity cost on the Spanish electricity under Phase II and III of the EU ETS and found that there were not only long-run equilibrium relations but also short-run interactions between the electricity price and the fuel and carbon price [31]. Woo et al. quantified the effect of California’s CO2 cap-and-trade program on the electricity prices in the western US, and the results showed that a $1/metric ton increase in California’s CO2 prices is estimated to have increased the electricity price [32]. Peña and Rodríguez studied the impact of renewables and other fundamental determinants on the electricity price in ten EU countries and found that the increase in production from renewables decreases wholesale electricity prices in all countries; however, it should promote electricity consumption [33]. Mosquera-López and Nursimulu took into account nonlinearities in electricity price in Germany and found that in the future market of electricity, the main drivers are natural gas, coal and carbon price [8]. Liu and Jin used a standard econometric approach analyzing the interactions between electricity, fossil fuel and carbon market prices in Guangdong, China, and they found that the electricity price was significantly and positively associated with coal price but had no significant relationship with carbon price and LNG price [34]. Biber et al. used logistic regression to study the influence of energy source, fuel, and emission price on electricity price and found that the volatile generation of wind solar power will raise the likelihood of low and negative electricity prices, and a higher CO2 allowance price will reduce the likelihood of negative prices [12].

Climate change has had a growing impact on human society in recent years; especially, extreme climate events have an important impact on energy demand, which in turn affects electricity prices. Santamouris et al. assessed the impact of the urban climate on the energy consumption of buildings in Athens and found that where the mean heat island intensity exceeded 10 °C, the peak electricity load for cooling purposes might be tripled [35]. Taseska et al. used a MARKAL Macedonia model to identify the interactions between climate change and the energy demand in Macedonia and proposed the electricity production structure and energy demand of three different climate change cases [36]. Mosquera-López et al. used an event study approach to study the unexpected spikes in electricity price and found that when a freezing event occurs, the average electricity price increases in the Nord Pool market [37]. Jasiński presented a way of creating three new variables based on air temperature to be used in forecasts of electricity price and found the new model can reduce the MAPE by up to 15.3% [38]. Guo et al. applied a TVP-VAR-SV model to analyze the nonlinear effects of climate policy uncertainty on global prices of crude oil and natural gas and found that responses of energy prices changed from positive to negative [39]. Ozturk et al. used a multivariate stochastic volatility model and found that climate uncertainty indeed serves as a significant driver of price fluctuations in emissions prices [40]. Lin et al. found that the extremely high price risk of electricity mainly occurred during severe icing intervals and was located in the regions away from major energy resources based on case studies [41].

The existing studies in the literature have carried out a lot of research on the relationship between electricity markets, other related markets and climate risks, while most of them support that energy prices, carbon prices and climate risks have a significant impact on electricity prices. However, most of the existing studies focus solely on the one-way impact, and they are mostly limited to the relationship between a small number of markets. Few studies discuss the transmission relationship of electricity prices between regions while comprehensively exploring the relationship between electricity prices, energy prices, carbon prices and extreme climate risks from a systematic perspective. Therefore, this paper will build a double-layer system based on a spillover effect using daily data. In the first layer, the spillover relationship between electricity prices in five typical European countries will be explored, and then in the second layer, the spillover effects among electricity price, oil price, natural gas price, carbon prices, carbon emissions, and extreme climate will be proposed.

## 3. Methodology and Datasets

### 3.1. Methodology

A double-layer spillover system is constructed in this paper; the spillover effects in each layer will be calculated based on the method proposed by Diebold and Yilmaz [42]. The first layer system examines the dynamic connectedness among spot electricity prices in five representative European countries, including the United Kingdom, Germany, Italy, Spain, and France. By constructing the first layer system, the spillover effects among different electricity markets in Europe can be analyzed. At the same time, based on the spillover network, indicators that reflect the systemic risk of the European electricity market can be constructed and used for construction of the second layer system. The second layer system takes into account the dynamic spillover effects among electricity price, crude oil prices, natural gas prices, carbon prices, *HDD* (Heating Degree Day), and *CDD* (Cooling Degree Day), and it also considers the systemic risk of electricity price fluctuations obtained in the first layer. Carbon emissions can reflect the energy structure, which will not only affect the price of carbon emissions but also influence the cost of other energy, thus affecting the prices of crude oil, natural gas and electricity. At the same time, climate risk will also indirectly affect other energy prices through its impact on carbon emission. So, carbon emission in Europe is also considered in the second-layer system.

Diebold and Yilmaz’s approach has been widely applied to the analysis of spillover effects among different markets [43,44]. The measurement of spillover effects is based on generalized Vector Autoregressive (VAR) models and Forecast Error Variances Decomposition (FEVD). The spillover effect among different variables can be derived from the FEVD of moving average representation of a VAR model.

The generalized VAR model with multiple stationary time series should be estimated first of all as shown in Equation (1).

(1) $$R_{t}=\sum_{i=1}^{p}\Phi_{i}R_{t-i}+\varepsilon_{t}$$

where $R_{t}$ is a vector of the endogenous variable in each layer, $\Phi_{i}$ are autoregressive coefficient metrices, $\varepsilon_{t}$ is the error vector, and *p* is the lag order, which can be determined by the HQ information criterion.

Then, the VAR model needs to be converted into the corresponding Vector Moving Average (VMA) model with infinity lag order, as shown in Equation (2).

(2) $$R_{t}=\sum_{j=0}^{\infty }A_{j}\varepsilon_{t-j}$$

where the coefficient matrix $A_{j}$ obeys a recursion of the form:

(3) $$A_{j}=\Phi_{1}A_{j-1}+\Phi_{2}A_{j-2}+\cdots +\Phi_{p}A_{j-p}$$

and $A_{0}$ is an identity matrix. Using the VMA format, the pairwise, directional and total connectedness can be measured based on the generalized FEVD approach. The *H* step-ahead generalized FEVD can be proposed, and the variance contribution of variable *j* to variable *i* can be obtained by Equation (4).

(4) $$\theta_{i,j}\left(H\right)=\frac{\sigma_{jj}^{-1}\sum_{h=0}^{H-1}{\left(e_{i}^{\prime }A_{h}\sum e_{i}\right)}^{2}}{\sum_{h=0}^{H-1}\left(e_{i}^{\prime }A_{h}\sum A_{h}^{\prime }e_{i}\right)}$$

where Σ is the variance matrix for the error vector ε and $\sigma_{jj}$ is the standard deviation of the error term of the $j^{th}$ equation. $e_{i}$ is a selection vector with a value of 1 for the $i^{th}$ element and 0 otherwise. Thus, the spillover index yields a matrix $\theta \left(H\right)=\theta_{i,j}\left(H\right)$. Then, each entry in the matrix can be normalized by the sum of each row.

(5) $${\tilde{\theta }}_{i,j}\left(H\right)=\frac{\theta_{ij}^{H}}{\sum_{j=1}^{N}\theta_{ij}^{H}}$$

Based on the normalized matrix $\tilde{\theta }$, some indicators can be obtained to investigate the spillover effects of each layer. The total spillover-to index $C_{i\to ∙,t}$ and total spillover-from index $C_{i\leftarrow ∙,t}$ of $i^{th}$ variable can be calculated, as defined in Equations (6) and (7).

(6) $$C_{i\to ∙,t}=\sum_{j=1,i\neq j}^{m}\varphi_{ji,t}^{-H}$$

(7) $$C_{i\leftarrow ∙,t}=\sum_{j=1,i\neq j}^{m}\varphi_{ij,t}^{-H}$$

Thus, a net spillover index $C_{i,t}$ of the $i^{th}$ variable can be calculated as shown in Equation (8).

(8) $$C_{i,t}=C_{i\to ∙,t}-C_{i\leftarrow ∙,t}$$

Furthermore, an aggregate index TCI measuring spillovers over the entire endogenous system can be constructed, as shown in Equation (9).

(9) $$TCI=\frac{1}{N}\sum_{i,j=1}^{N}\theta_{i,j},\;j\neq i$$

where *N* is the number of the endogenous variables in each layer.

Referring to Diebold and Yilmaz (2012), the forecast horizon *H* is setting to integers between 5 and 15, respectively, and the results are robust. In the empirical analysis section, the results with forecast horizon *H* = 10 are showed as a representation.

In order to obtain the dynamic spillover effects, the w-day rolling windows method is used with 1 day scrolling forward each time. In order to minimize the loss of sample size, in the dynamic analysis of the first layer system, a 100-day rolling window is used. A 200-day rolling window is adopted in the second layer system, as the model has more variables. Furthermore, 100-day, 150-day and 200-day rolling windows are all used in each layer to check the robustness of the results.

### 3.2. Data Description

#### 3.2.1. Electricity Prices

**Figure 1:**
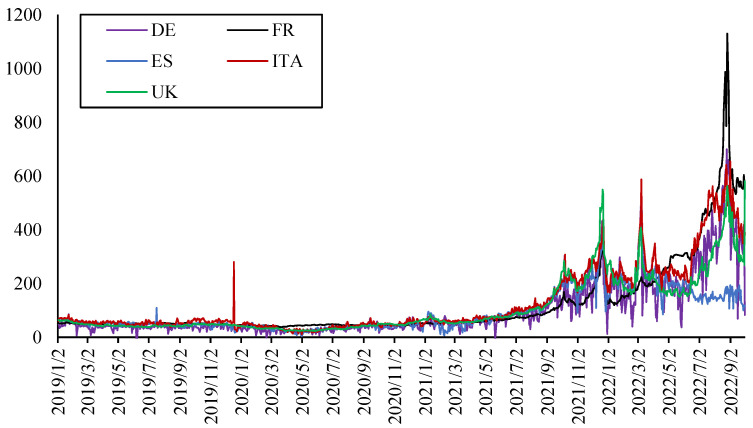
Electricity prices in five European countries.

The data of electricity prices used in this paper are spot benchmarks offered by sellers to buyers priced in megawatts per hour (MWh) in the local currency of each country. To study the connectedness of electricity prices in different countries in Europe, five representative countries were selected, including the United Kingdom (UK), Germany (DE), Italy (ITA), Spain (ES), and France (FR). In order to ensure the sample interval of the electricity spot price data coverage range is consistent with the indicators in the second layer system, the daily data from 1 January 2019 to 30 September 2022 are used for empirical analysis. The dataset is from the Trading Economics. Non-trading days are deleted. Furthermore, some missing values are processed using a linear interpolation. As shown in Figure 1, the electricity prices in all five countries have been rising rapidly and fluctuating sharply since the Russia–Ukraine conflict, especially those in France, which once exceeded €1000 per megawatt-hour (MWh). The fluctuation trend of the electricity prices in all countries is consistent before 2022, while the electricity price of Spain in 2022 is different from that of the other four countries, remaining relatively stable. It mainly due to the electricity supply structure of various countries. Spain has a relatively high proportion of nuclear, wind and hydro electricity generation, so its electricity price remains relatively stable.

To ensure the stationary of the data used for modeling, the daily return of each electricity price is calculated. The descriptive statistics and stationary tests are shown in Table 1. It can be found that the average electricity returns of the five countries are all greater than 0, among which the highest average daily growth rate in Germany is 4.31%, while its volatility is also the highest among the five countries. Meanwhile, the results of the ADF test showed that all the data are stationary. The results of the correlation analysis are shown in Table 2; the electricity return in France and Britain, Spain and Italy show a significant positive correlation.

#### 3.2.2. Indicators of Other Markets

**Figure 2:**
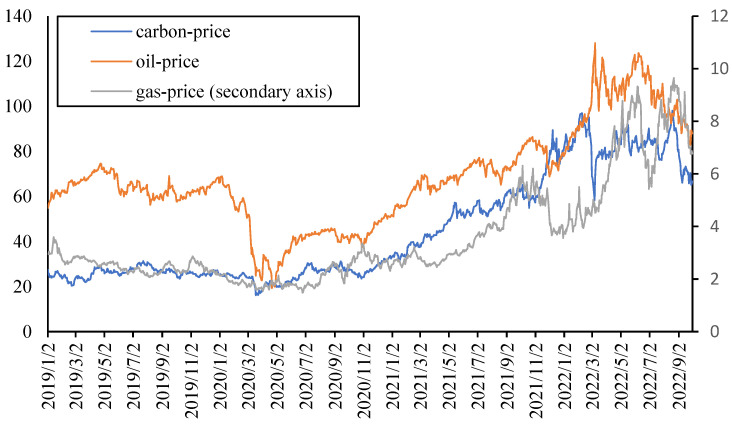
Crude oil price, natural gas price and carbon price.

In order to explore the spillover effects among European electricity prices and other markets, this paper also considers the crude oil price (OIL), natural gas price (GAS), carbon price (CARBON), and carbon emission (EMISSION). The daily data of London Brent Crude oil future price, natural gas future price and EU carbon emissions allowances future price are used, which is sourced from investing.com. Furthermore, the daily carbon emission data of Europe are obtained from carbonmonitor.org. As shown in Figure 2, the overall trend of the three prices remains consistent.

**Figure 3:**
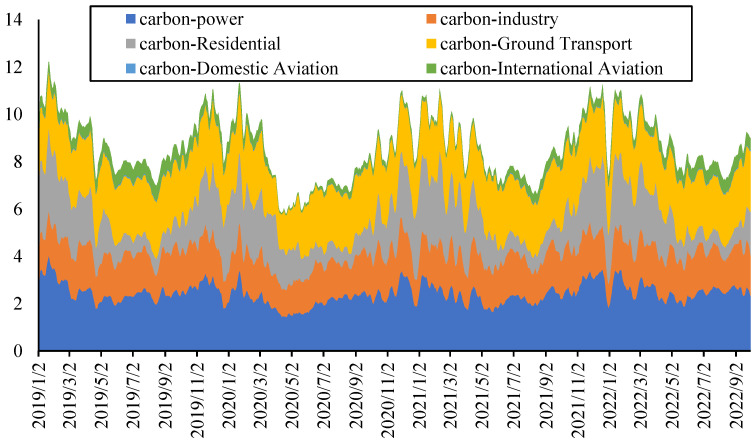
Carbon emissions from different departments.

The carbon emissions from different departments are shown in Figure 3. The overall trend and periodicity of carbon emissions of these departments are basically consistent except for the carbon emission from the international aviation department, while the proportion of carbon emissions from the international aviation department is relatively small. The total carbon emission of Europe is used in the modeling process.

The daily return of each indicator is also calculated for modeling. The descriptive statistics and stationary tests of these returns as well as average electricity prices (ELEC) are shown in Table 3. The mean of most indicators is greater than 0; that is, the average daily return is positive, among which the largest average daily return is the electricity price, which is followed by the carbon price. The most volatile is oil prices, which is followed by natural gas prices. At the same time, all the indicators are stationary based on the ADF test and can be directly used for modeling.

#### 3.2.3. Indicators of Climate Risk

The *HDD* (heating degree day) and *CDD* (cooling degree day) in Europe are constructed to measure extreme temperatures in this region. The degree day reflects the demand for energy to heat or cool houses and businesses. These two indicators are derived from daily temperature at the major weather station in the five countries. The daily temperatures of 15 °C and 22 °C are the basis for the heating and cooling degree day computations [45]. If the temperature is lower than 15 °C or higher than 22 °C, heating or cooling is required. Heating degree days are summations of negative differences between the mean daily temperature and the 15 °C base, as shown in Equation (10).

(10) $$HDD_{t}=\sum_{i=1}^{n}w_{i}∙\mathrm{Max}\left\{15\;℃-T_{i,t},\;0\right\}$$

where $T_{i,t}$ indicates the daily average temperature of weather station *i* in day *t*, and $w_{i}$ is the weight of station *i*. In this paper, the equal weight is used with $w_{i}=1/n$.

The cooling degree days are summations of positive differences from the 22 °C base.

(11) $$CDD_{t}=\sum_{i=1}^{n}w_{i}∙\mathrm{Max}\left\{T_{i,t}-22\;℃,\;0\right\}$$

If the mean daily temperature is higher than 15 °C, the *HDD* is zero, while if the mean daily temperature is lower than 22 °C, the *CDD* is zero. The original daily temperature data of the weather stations are sourced from NOAA (National Oceanic and Atmospheric Administration).

**Figure 4:**
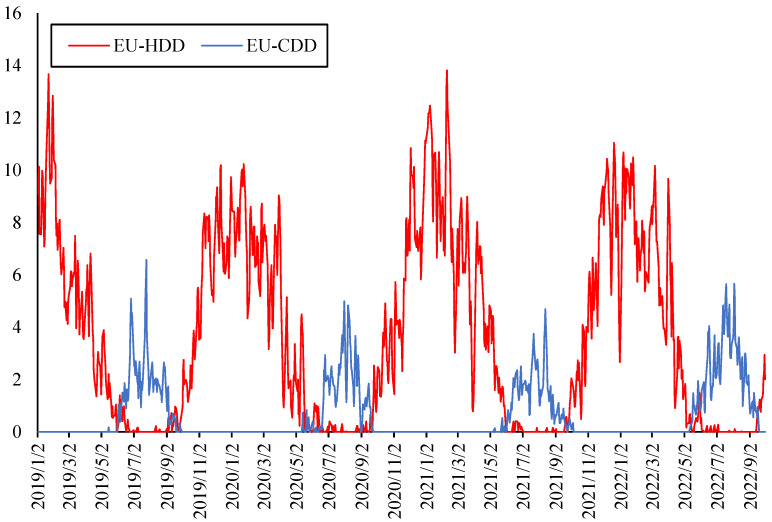
*HDD* and *CDD* in Europe.

Most parts of Germany, Britain and France have a temperate maritime climate, cool in summer and mild in winter, with a small annual temperature difference; however, northern Spain is cold in winter, while Italy has a Mediterranean climate with mild in winter, but it is hot in summer. As the *HDD* and *CDD* trends of these countries are basically consistent and the correlation coefficient between either the *HDD* or *CDD* of each pair of countries is relatively high, the average values of *HDD* and *CDD* in each weather station are taken as the *HDD* and *CDD* in Europe, respectively. As shown in Figure 4, the *HDD* is higher than the *CDD*, but the annual average value of *CDD* tends to increase in the last three years. Since neither *HDD* nor *CDD* are stationary series, the first difference indicators *D_HDD* and *D_CDD* are used, which are stationary and based on the ADF test.

The correlation matrix of all the indicators in the second layer is shown in Table 4, *HDD* has a strong positive correlation with carbon emissions, and there are also certain positive correlations between crude oil return and carbon return, between electricity return and carbon emissions, and between crude oil return and natural gas return.

## 4. Connectedness among Electricity Prices in the European Countries

### 4.1. Static Analysis of the Full Sample

The static spillover matrix of the full sample is shown in Table 5; the UK electricity return has the largest net directional connectedness, which is followed by Germany. The net directional connectedness of France, Spain and Italy is negative; that is, they are mainly affected by electricity returns of other countries. The spillover effects between Britain and France are higher, which is consistent with the conclusion of the previous correlation analysis. It is mainly because the UK is a major importer of electricity in Europe, while Germany has the largest net electricity exports.

### 4.2. Dynamic Analysis of Connectedness among Electricity Prices

**Figure 5:**
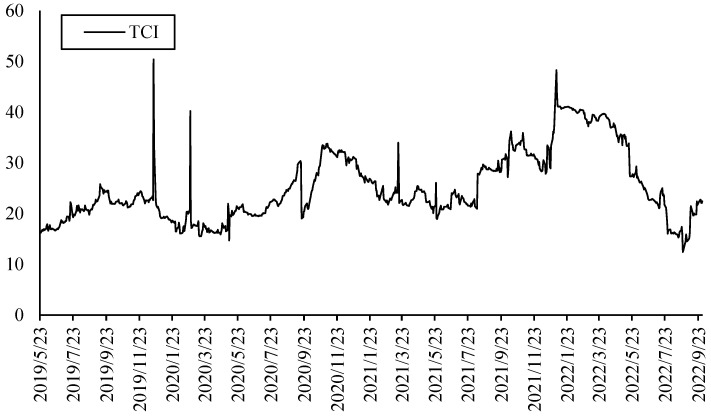
TCI of the first layer (five electricity markets).

Figure 5 shows the TCI of the first layer which presents the overall connectedness or systemic risk of electricity return in the five countries. It can be found that the overall connectedness is relatively stable. However, since the beginning of the Russia–Ukraine conflict, the connectedness had increased to a certain extent. Due to the large differences in the electricity supply and consumption structure of different countries, even though the electricity price of all countries generally increases driven by the high natural gas price, the connectedness has gradually recovered to the level before the Russia–Ukraine conflict.

**Figure 6:**
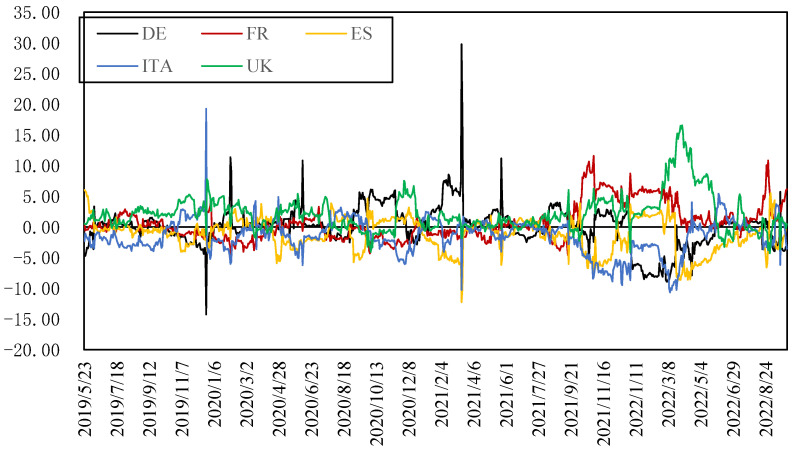
Net spillover indexes of five electricity returns.

Figure 6 shows the dynamic net spillover index of electricity return in each country. Before the Russia–Ukraine conflict, the dynamic net spillover situation of various countries remained relatively stable. However, since the Russia–Ukraine conflict, this stable relationship has been broken, and the electricity returns of the UK and France show a relatively significant positive net spillover effect. Because the UK is a major importer of electricity and mainly relies on natural gas in its electricity generation, it plays a leading role in this round of electricity price rise; France relies on nuclear generation, so its electricity supply is relatively stable and less affected by other markets.

## 5. Spillover Effects among Markets under Climate Change

### 5.1. Static Analysis of the Full Sample

**Figure 7:**
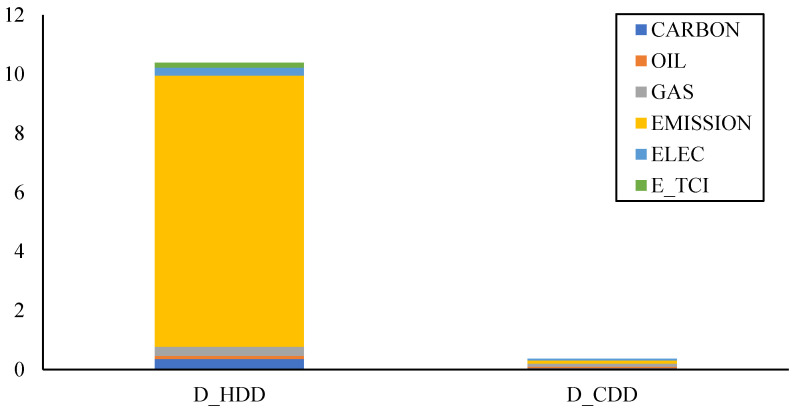
Spillover effects from *D_HDD* and *D_CDD* to other indicators.

The static spillover matrix of the full sample is shown in Table 6. In addition to the average electricity price in Europe, the systemic risk in the electricity market indicated by the TCI (E_TCI) in the first layer is also taken into account. The carbon emission has the highest net directional connectedness, which is followed by the electricity prices and systemic risk of electricity markets. Carbon emission has a high spillover effect on both the electricity price and the systemic risk of electricity market, with a directional connectedness index of 1.12 and 0.62, respectively. *D_HDD* has a high level of directional connectedness to others, while *D_CDD* almost has no spillover effects on other indicators. The spillover effects from *D_HDD* and *D_CDD* to other indicators can be seen in Figure 7. The spillover effect index from *D_HDD* to carbon emission is as high as 9.17, far exceeding the spillover effects on other markets. At the same time, the spillover effect from *D_HDD* on carbon price, natural gas price and electricity price is relatively high. It is due to the heating degree for low-temperature increased demand for energy, which increasing the level of carbon emission and energy prices. However, due to the relatively suitable summer temperature in most areas of Europe, the spillover effect from *D_CDD* to various markets is relatively small.

### 5.2. Dynamic Analysis of Spillover Effects among Markets under Climate Change

**Figure 8:**
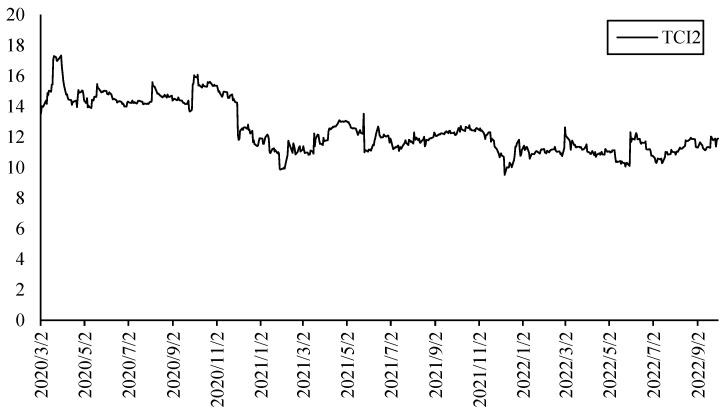
TCI of the second layer (different markets under climate risks).

Figure 8 shows the TCI of the second layer which presents the overall connectedness between electricity and other markets considering climate factors. It can be found that the overall connectedness is more stable than that of the first layer. It only had a significant increase in the early days of the COVID-19, but it began to stabilize at around 10 levels in the fourth quarter of 2020. It is not affected by the Russia–Ukraine conflict and the sharp rise in energy prices.

**Figure 9:**
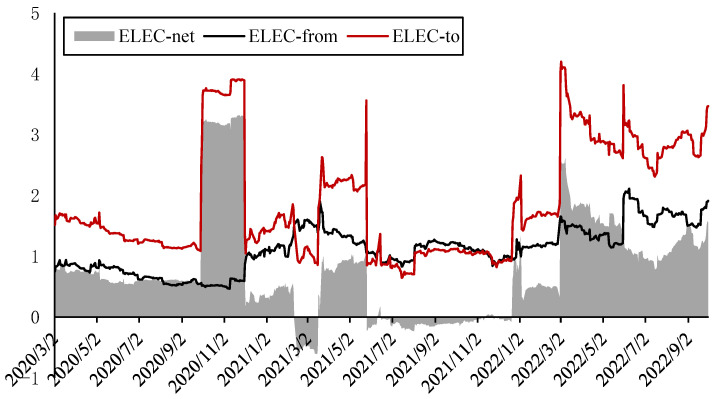
Dynamic spillover effects of electricity return.

**Figure 10:**
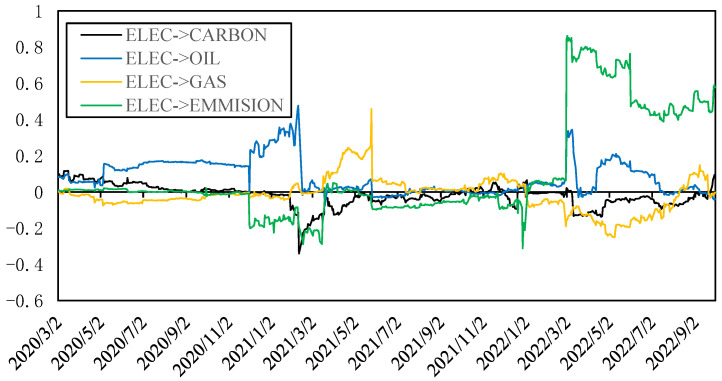
Dynamic net spillover effects from electricity return to other indicators.

Figure 9 shows the dynamic spillover effects of electricity return. It can be found that the total spillover effects from electricity return to other indicators (ELEC-to) is usually greater than the total spillover effects from other indicators to it (ELEC-from), so the net spillover effect (ELEC-net) is positive in most stages. Among them, only within six months after the beginning of the Russia–Ukraine conflict, ELEC-to and ELEC-from were roughly equal. The ELEC-from is relatively stable. However, ELEC-to shows large fluctuations; in the fourth quarter of 2020, the second quarter of 2021 and the second quarter of 2022, ELEC-to has a larger rise. From the structure of spillover effects, the ELEC-to increased in these stages mainly due to pulling up of the spillover effect from the electricity return to the systemic risk of the electricity market as the electricity price rises rapidly simultaneously. After eliminating the spillover effect from the electricity return to the systemic risk of the electricity market, the net spillover effects structure of the electricity return is shown in Figure 10. It can be found that the electricity return had a positive net spillover effect on the crude oil return before 2021, but the net spillover was reduced to almost 0 after the Russia–Ukraine conflict, while it had a positive spillover effect on the natural gas return in the short term. In 2022, the electricity supply from renewable energy sources in Europe is tight again; the electricity return showed a high spillover effect on carbon emission and crude oil return, but at the same time, the net spillover effect on natural gas and carbon price is negative: that is, there are net spillover effects from natural gas and carbon prices to electricity price, which are also the main factors of the electricity price increase in 2022.

**Figure 11:**
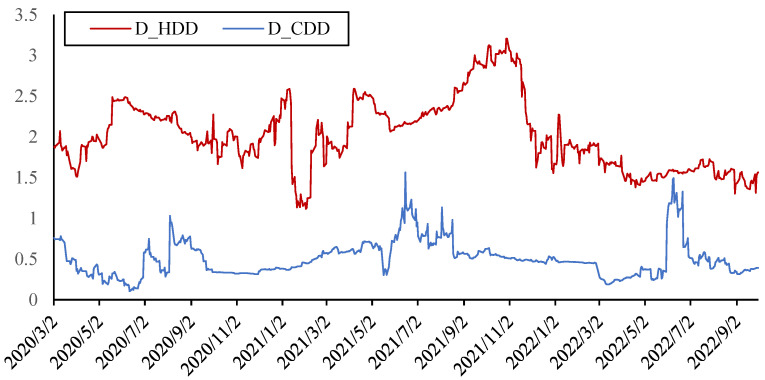
Dynamic spillover effects from *HDD* and *CDD* to other indicators.

Dynamic spillover effects from *HDD* and *CDD* to other indicators are shown in Figure 11. The results also proved that under the climate characteristics of Europe, the impact of *HDD* on the energy markets is much greater than that of *CDD*. The heating demand has a significant impact on all kinds of energy prices and carbon emission, while this spillover effect will further rise in the extreme cold stage. At the same time, as the summer temperature is not high in most areas of Europe, the cooling demand is relatively low. It can be seen in Figure 11 that the spillover effect from *CDD* to other indicators is relatively small, only showing a temporary rise in a short period of summer. However, due to global warming, the overall trend of *HDD* spillover effect did not rise, while the *CDD* spillover effect showed a certain upward trend.

## 6. Conclusions

A double-layer system based on spillover effects using daily data of electricity prices as well as other related indicators is constructed in this paper while considering the impact of climate risks. The first layer system mainly considers the spillover effects among electricity prices in five European countries including the United Kingdom, Germany, Italy, Spain, and France; the second layer system mainly considers the spillover effects among electricity price, crude oil price, natural gas price, carbon price and carbon emission, as well as the climate risk factors including heating degree day and cooling degree day.

The results of the first layer system based on the electricity markets of different European countries show that there are certain spillover effects between electricity prices, among which the UK has the largest net directional connectedness, which is followed by Germany. The importance of the electricity market is mainly related to the status of the electricity trade. The dynamic analysis shows that the overall spillover index remains relatively stable, with only a temporary rise at the beginning of the Russia–Ukraine conflict.

The second layer system which focuses on the spillover effects among the electricity market and other energy-related markets considering climate risk shows that the electricity market is not only affected by crude oil, natural gas and carbon markets but also has a feedback effect to these markets. The results of the static analysis show that the price and systemic risk of the electricity market have a relatively larger net directional connectedness that is only smaller than carbon emission; that is, there is a significant spillover effect from the electricity market to other markets. The results of the dynamic analysis further indicate that there are spillover effects from the electricity market to other markets in most of the range. Especially since 2022, since the renewable energy generation cannot fully meet the electricity demand of Europe, the electricity market has a large spillover effect on carbon emission. At the same time, climate risks also have an important impact on the European energy system including the electricity market, especially the heating degree day. The demand for heating will affect the prices in various energy markets as well as carbon emission by driving energy consumption.

This paper systematically analyzes the spillover effects among the European electricity markets and those with other related energy markets while innovatively introducing climate risks into this system. On the one hand, it reveals the important role of the electricity market in the European energy market. On the other hand, it also confirms the significant impact of climate risks on energy prices. In the case of increasing geopolitical risks in the world and frequent energy crises, understanding the connectedness among various markets and the impact of climate risks has a certain guiding significance for better preventing market price risks and reasonable hedging the risks.

## Figures and Tables

**Table 1:** Descriptive statistics and stationary tests of electricity return.

	FR	ES	DE	ITA	UK
Mean	0.0030	0.0191	0.0431	0.0133	0.0039
Median	0.0013	0.0038	−0.0235	0.0000	0.0004
Maximum	0.2614	2.6986	5.8634	3.9831	1.1536
Minimum	−0.3588	−0.8372	−16.9603	−0.8164	−0.3593
Std. Dev.	0.0341	0.2173	0.8445	0.1890	0.0612
Skewness	−0.9445	4.7766	−8.4192	10.7970	7.0598
Kurtosis	33.7229	52.1793	195.5536	212.9305	134.5466
Jarque–Bera	38,214.41	101,231.50	1,506,870.00	1,796,330.00	705,989.50
Probability	0.0000	0.0000	0.0000	0.0000	0.0000
ADF t-Statistic	−15.0695	−13.2338	−32.0745	−14.6691	−25.4600
Probability	0.0000	0.0000	0.0000	0.0000	0.0000
Observations	968	968	968	968	968

**Table 2:** Correlation matrix of electricity return.

	FR	ES	DE	ITA	UK
FR	1.0000	−0.0145	0.0302	0.0107	0.3412
ES	−0.0145	1.0000	−0.0037	0.2282	0.0133
DE	0.0302	−0.0037	1.0000	0.0123	−0.0001
ITA	0.0107	0.2282	0.0123	1.0000	0.0222
UK	0.3412	0.0133	−0.0001	0.0222	1.0000

**Table 3:** Descriptive statistics and stationary tests of other market-indicators.

	OIL	GAS	CARBON	EMISSION	ELEC
Mean	0.0010	0.0000	0.0013	0.0003	0.0165
Median	0.0031	0.0000	0.0019	0.0005	0.0044
Maximum	0.2102	1.9556	0.1751	0.1204	1.1417
Minimum	−0.2440	−3.2444	−0.1625	−0.1612	−3.3717
Std. Dev.	0.9297	0.4236	0.0294	0.0243	0.1818
Skewness	−0.6947	−1.2095	−0.2621	−0.2434	−6.6101
Kurtosis	17.0890	15.3959	6.9397	9.2374	146.1563
Jarque–Bera	8083.97	6433.51	637.12	1578.74	833,629.80
Probability	0.0000	0.0000	0.0000	0.0000	0.0000
ADF t-Statistic	−28.8675	−32.1444	−33.9706	−17.0033	−31.7333
Probability	0.0000	0.0000	0.0000	0.0000	0.0000
Observations	968	968	968	968	968

**Table 4:** Correlation matrix of all the indicators in the second layer system.

	OIL	GAS	*D_HDD*	*D_CDD*	ELEC	CARBON	EMISSION
OIL	1.0000	0.1065	−0.0234	0.0035	0.0148	0.1950	0.0474
GAS	0.1065	1.0000	−0.0499	0.0066	0.0684	0.0868	−0.0046
*D_HDD*	−0.0234	−0.0499	1.0000	0.0019	0.0299	−0.0316	0.3197
*D_CDD*	0.0035	0.0066	0.0019	1.0000	−0.0184	−0.0113	−0.0074
ELEC	0.0148	0.0684	0.0299	−0.0184	1.0000	0.0260	0.1127
CARBON	0.1950	0.0868	−0.0316	−0.0113	0.0260	1.0000	−0.0737
EMISSION	0.0474	−0.0046	0.3197	−0.0074	0.1127	−0.0737	1.0000

**Table 5:** The static spillover matrix of electricity prices.

Item	DE	FR	ES	ITA	UK	FROM
DE	98.84	0.85	0.01	0.16	0.13	1.16
FR	0.17	85.03	0.74	0.42	13.63	14.97
ES	0.62	0.85	92.81	4.94	0.78	7.19
ITA	0.74	0.64	4.34	92.44	1.85	7.56
UK	0.02	10.88	0.14	0.11	88.85	11.15
Directional TO Others	1.56	13.22	5.22	5.63	16.40	42.03
Directional Including Own	100.40	98.26	98.03	98.07	105.24	TCI
NET Directional Connectedness	0.40	−1.74	−1.97	−1.93	5.24	8.41

**Table 6:** The static spillover matrix of different markets under climate change.

Item	*D_HDD*	*D_CDD*	CARBON	OIL	GAS	EMISSION	ELEC	E_TCI	FROM
*D_HDD*	88.38	0.01	0.28	0.22	0.40	10.30	0.00	0.41	11.62
*D_CDD*	0.01	99.76	0.04	0.00	0.01	0.03	0.07	0.09	0.24
CARBON	0.36	0.05	93.25	4.09	0.91	0.71	0.20	0.42	6.75
OIL	0.11	0.05	4.12	93.06	1.44	0.25	0.67	0.31	6.94
GAS	0.31	0.11	1.04	1.39	95.87	0.15	0.51	0.62	4.13
EMISSION	9.17	0.10	0.06	0.20	0.40	88.32	1.12	0.62	11.68
ELEC	0.27	0.06	0.12	0.03	0.66	1.29	97.42	0.15	2.58
E_TCI	0.16	0.00	0.02	0.29	0.09	0.70	0.85	97.88	2.12
Directional TO Others	10.39	0.39	5.67	6.22	3.90	13.43	3.43	2.63	46.06
Directional Including Own	98.77	100.14	98.92	99.27	99.77	101.75	100.85	100.51	TCI
NET Directional Connectedness	−1.23	0.14	−1.08	−0.73	−0.23	1.75	0.85	0.51	5.76

## Data Availability

The data can be provided for requirement.
